# The Social Meaning of Contextualized Sibilant Alternations in Berlin German

**DOI:** 10.3389/fpsyg.2020.566174

**Published:** 2020-10-29

**Authors:** Melanie Weirich, Stefanie Jannedy, Gediminas Schüppenhauer

**Affiliations:** Leibniz-Centre General Linguistics, Berlin, Germany

**Keywords:** sociophonetics, perception, social meaning, social context, IAT, fine phonetic detail, prestige, in-group – out-group

## Abstract

In Berlin, the pronunciation of /ç/ as [ɕ] is associated with the multi-ethnic youth variety (*Kiezdeutsch*). This alternation is also known to be produced by French learners of German. While listeners form socio-cultural interpretations upon hearing language input, the associations differ depending on the listeners’ biases and stereotypes toward speakers or groups. Here, the contrast of interest concerns two speaker groups using the [ç]–[ɕ] alternation: multi-ethnic adolescents from Berlin neighborhoods carrying low social prestige in mainstream German society and French learners of German supposedly having higher cultural prestige. To understand the strength of associations between phonetic alternations and social attributes, we ran an Implicit Association Task with 131 participants (three groups varying in age and ethnic background (mono- vs. multi-ethnic German) using auditory and written stimuli. In experiment 1, participants categorized written words as having a positive (*good*) or negative (*bad*) valence and auditory stimuli containing pronunciation variations of /ç/ as canonical [ç] (labeled *Hochdeutsch* [a term used in Germany for Standard German]) or non-canonical [ɕ] (labeled *Kiezdeutsch*). In experiment 2, identical auditory stimuli were used but the label *Kiezdeutsch* was changed to *French Accent*. Results show faster reaction times when negative categories and non-canonical pronunciations or positive categories and canonical pronunciations were mapped to the same response key, indicating a tight association between value judgments and concept categories. Older German listeners (OMO) match a supposed *Kiezdeutsch* accent more readily with negatively connotated words compared to a supposed French accent, while younger German listeners (YMO) seem to be indifferent toward this variation. Young multi-ethnic listeners (YMU), however, seem to associate negative concepts more strongly with a supposed French accent compared to *Kiezdeutsch*. These results demonstrate how social and cultural contextualization influences language interpretation and evaluation. We interpret our findings as a loss of cultural prestige of French speakers for the YMO group compared to the OMO group: younger urban listeners do not react differently to these contextual primes. YMU listeners, however, show a positive bias toward their in-group. Our results point to implicit listener attitudes, beliefs, stereotypes and shared world knowledge as significant factors in culturally- and socially situated language processing.

## Introduction

In this study, we will show that listeners draw implicit associations between sub-phonemic variation or fine phonetic detail and evaluative categories in dependence to a speaker group that supposedly produced the speech form. Speech production is not merely a means of transporting propositional content, but also serves the construction of personas and reflects speakers’ social identities. For simplicity reasons, here in this paper we use the binary concept of canonical vs. non-canonical, however, our conceptualization of individuals’ speaking styles goes beyond the notion of complementary, binary or dichotomous categories such as formal vs. informal or casual, standard vs. non-standard, or read vs. spontaneous. Rather, we conceive speech with all its features and variants as a tool set from which speakers (sub)consciously select and chose from to position themselves in social space. Listeners pay attention to phonetic detail and either can or cannot interpret the social meaning of the variant(s).

However, many of the fine phonetic details observed in speech are produced by a speaker without much awareness. As the social dynamics change or the persona performed varies by situation or shifts over the course of a conversation, so too can the linguistic choices of the speaker. Hearers may then be in a position to draw meaningful associations between linguistic variants and social actors that use variants to create a personal style or to index a particular social persona, properties or stances. Speech researchers have long come to the realization that speakers adjust their speech in dependence to language external factors (Labov, 2001) such as the addressee (Bell, 1984), specifically in child- or animal directed speech (Burnham et al., 2002), speaker characteristics such as age (Eckert, 1989), gender identity (Weirich and Simpson, 2018), sexual orientation (Munson et al., 2006; Kachel et al., 2018), or the formality of the speech situation (Podesva, 2007). Also, a speaker’s phonetic accommodation to a model talker is mediated by the other speaker’s social identity and perceived attractiveness (Babel, 2012) or the participants personality (Lewandowski and Jilka, 2019). The degree of convergence has been found to be used to decrease or increase social distance (Giles, 1973; Giles et al., 1973; Bourhis and Giles, 1977). A speaker’s perceived femininity or masculinity plays a role in perception (Johnson et al., 1999) as does a hearer’s age (Jannedy and Weirich, 2014) or where the hearer believes the speaker is from Niedzielski (1999), Hay and Drager (2010), Jannedy and Weirich (2014).

The study of intra-speaker variation as a field of study has gained traction with the *Third Wave* in sociolinguistics (Eckert, 2012; Eckert and Labov, 2017) where studies focus on the speech styles of individuals as they maneuver social situations. While phonetic variation is inherently gradient, the occurrence of a phonetic form can statistically be used more often in one social situation or by one social group compared to another. Eckert and Labov (2017, p. 481) explicitly say that “Having no referential function, a phonological variable is free to take on purely contextual meaning as it ranges within the limits set by neighboring phonemes.” So, once a variant has been collectively recognized by listeners as belonging to a specific speech style, context, or social group, it can be used to index membership in this group or to index a specific context.

For example, in German, the phonological category /ç/ has two allophonic variants (throughout this manuscript we will refer to the default or canonical German fricative variant as [ç] and to the non-canonical alternant as [ɕ]). The alternation of /ç/ to a phonetic variant ranging acoustically between the palatal fricative [ç] and the post-alveolar fricative [ʃ], i.e., symbolically represented as the alveopalatal fricative [ɕ] in the youth-style multi-ethnolect Kiezdeutsch as spoken in Berlin – the sociolect investigated in the present study – serves to index membership and the identification with the multi-cultural Berlin districts Kreuzberg, Neukölln or Wedding as “their” neighborhood in the speech of adolescents (Jannedy et al., 2015). As an extension to that, for some people, it indexes a young, hip, multiethnic and urban *street* identity. For simplicity reasons, we will refer to this variant of /ç/ as [ɕ] as our work on the acoustic phonetic properties of these variants suggests that [ɕ] differs from both [ç] and [ʃ] in several spectral parameters such as center of gravity (COG, cf. section “Acoustic Characteristics of Stimulus Materials”) and discrete cosine transformation coefficients (Jannedy and Weirich, 2017).

Work on language stereotypes, attitudes (Johnson et al., 1999; Niedzielski, 1999; Hay and Drager, 2010; Jannedy and Weirich, 2014), and person perception (Scherer, 1972; Schirmer, 2019) has shown that it is possible to put speakers in mind-sets in which to perceive speech. We will exploit this finding for our study, too by making listeners believe that a voice they hear either belongs to a French speaker learning German or a German speaker of Turkish decent, both groups for which stereotypes exist in dominant German language ideology (Plewnia and Rothe, 2009; Jannedy and Weirich, 2014; Jannedy et al., 2019). While German spoken with a French accent supposedly is the most favored foreign accent by Germans and generally evokes positive ratings (Plewnia and Rothe, 2009), German spoken with features believed to be of multi-ethnic origin, i.e., Arabic or Turkish seems to polarize or evoke negative stereotypes (Wiese, 2015). It is our assumption that neither the positive nor the negative associations with these two varieties of German are conscious so as to be deliberately mediated in public, and moreover, vary between individuals influenced by social factors such as age or personal background.

In this work, we investigate the relative strength of implicit associations between speech variants and evaluative categories in the context of a fictitious French vs. multi-ethnic speaker group. We have borrowed the experimental technique of the Implicit Association Task (IAT) (Greenwald et al., 1998, 2003) from psychology as we are interested in the immediate and unmediated reactions to a speech stimulus and the social information that a phonetic shape invokes. According to the *Social Connotation Hypothesis* (van Bezooijen, 2002), hearers’ evaluations and reactions to language stimuli depend on social attributes and inferences drawn based on the supposed values, intentions, and attitudes that are associated with a speech variant. The IAT paradigm allows for collecting reaction time data which reveals how strongly a listener associates a specific variant with a value judgment. Due to the structure of the task, participants should not be able to disguise which associations come closest to their own, thus revealing their implicit, rather than their explicit, associations.

There is much evidence that encountered language input is stored in memory along with social information. These remembered instances (“exemplars”) are stored in a multi-dimensional space representing a cognitive map (Goldinger, 1998; Barlow and Kemmer, 2000). Watanabe et al. (2001) stipulate that the human cognitive system is built in such a way that learning in general and by extension perceptual learning works with and without awareness through rapid adaptation to the surrounding environment. This is also corroborated by the work on sound acquisition and acquisition trajectories (Foulkes and Docherty, 2006), and sound change (Harrington, 2006). Harrington et al. (2019) for example showed that during the linguistic isolation of multi-dialectal English-speaking staff during the winter months in Antarctica, their speech begins to converge toward each other, averaging out differences in vowel production (also see Eckert, 2019 on the spread of sound change). Results like these imply that groups of speakers that have a sense of belonging to the same social group and probably identifying with it, may develop speech patterns that can eventually be interpreted in meaningful ways by hearers. Applying this train of thought to our study, we are interested in the associated information that is stored with a phonetic variant in the context of two distinct speaker groups and the way associated and implied social information shapes the attitudes associated with specific speech forms.

An example widely discussed in the literature (Campbell-Kibler, 2011, 2012 and references therein) is the English verbal suffix <*-ing>* which is realized as either [ɪŋ̜] with a velar nasal in many standard varieties of English or as [ɪŋ̜] with an alveolar nasal in non-standard varieties. It is argued that the choice of this variant by a speaker in speech production has a communicative intention (see Eckert, 2008; Campbell-Kibler, 2011; Eckert, 2012 and others). The work by Campbell-Kibler (2010; 2011; 2012) shows that addressees derive social associations such as *educated* or *intelligent* from speech variants, yet, these interpretations are highly context-sensitive and dependent on a listener’s mood and the social perception of the speaker (Campbell-Kibler, 2008).

Our own work (Jannedy and Weirich, 2014) on the [ç] – [ɕ] alternation in the urban context of Berlin revealed an age-graded listener bias in the categorization of stimuli taken from a 14-step acoustic continuum ranging from /ç/ to /ʃ/ (where [ɕ] is located along the continuum) when co-presented with the name of a Berlin neighborhood (*Kreuzberg*) known for its multi-ethnic and multi-lingual population. In this classic categorical perception task, older (mean age: 50.7) and middle (mean age: 30.2) aged listeners were biased in their responses toward the non-canonical pronunciation variant [ɕ] in the context of the prime *Kreuzberg* (the multi-ethnic and multi-lingual Berlin district), whereas younger (mean age: 22.7) listeners seemed to have been free of or have undone this bias by selecting fewer [ɕ] tokens in this condition compared to a control condition where no additional information was presented.

These results suggest that listeners have conceptualized representations or stereotypes of what people from certain neighborhoods sound like, affectively reacting to perceptual stereotypes and creating perceptual personas. Our results corroborate findings on the social association of the *-in/-ing* alternation in American English (Campbell-Kibler, 2010, 2011) whereby the canonical *-ing* pronunciation was associated with more intelligent/educated/articulate speaker types, while the *-in* pronunciation was perceived to sound less formal and less likely to be gay.

Results like these show that listeners tie pronunciation variants to social attributes, a connection that is undoubtedly learned. Studies show that listeners were not able to distinguish between the standard- and non-standard varieties of languages that were unknown to them (Giles et al., 1974, 1975; van Bezooijen, 1988) in terms of the perceived pleasantness or status, showing that there is no inherent value to one form over another. In other words, one variant is not more sophisticated than another variant, it is the implicit association of speech variants with assumed, associated or stereotyped social traits of speaker groups that lets members of a speech community form value judgments (cf. *Social Connotation Hypothesis*, van Bezooijen, 2002).

To gain a better understanding of these deeply rooted implicit associations that listeners have formed on variable speech production patterns and linked to learned, assumed or stereotyped social traits, we have used a method that measures the relative strength of association between two dichotomous concept categories. The IAT (Greenwald et al., 1998; Greenwald et al., 2003; Nosek et al., 2005) investigates the immediate and affective inferences that participants draw upon being prompted with (a set of) stimuli. Classic IAT experiments were used to show the closeness of implicit associations between concepts such as *male/female* and *science/humanities*, *black/white* and *good/bad*, or *skinny/fat* and *good/bad*. This experimental paradigm has also proven to be quite valuable for testing the tight association of phonetic forms with social meanings (see Campbell-Kibler, 2012). Campbell-Kibler (2012) has used the paradigm to show that experiment participants had an awareness of the -*ing/-in* variable when they were presented with it in writing, associating these forms with either professions (*white-collar/blue-collar*) or with regional accents (*southern/northern states*).

Pantos (2010) pioneered a multi-modal IAT-approach, presenting auditory and visual stimuli and a combination thereof. In follow-up work, Pantos and Perkins (2012) tested in an United States-American context the implicit association between pronunciation variants and positive and negative valence words and found an implicit bias in favor of United States-accented versus Korean-accented speech. Campbell-Kibler (2012) also successfully deployed an auditory paradigm and was able to show that the *-ing/-in* variables were implicitly associated with *northern* vs. *southern* accented speech, respectively. Nilsson et al. (2019) tested the differences in social meaning of Swedish /i/ in two rural areas. In one of their two test-sites, their results revealed a stronger implicit association of “damped” /i/ ([ɨ]) with ruralness and cardinal /i/ with urbanity.

In this work, we will test the implicit associations of words ending in the German adjectival suffix <*-ig>* (produced with the German palatal fricative [ç] varying in pronunciation between non-canonical [ɕ] and canonical [ç]) and positive and negative valence words (as representatives of positive and negative attitudes toward the variants and the speaker group using this variant).

Our first hypothesis is, that listeners will associate non-canonical pronunciations with negative values and canonical pronunciations with positive values. The additional aim is to exploit the similarity of the [ɕ] variant in the two distinct varieties of German *Kiezdeutsch* and *French Learners’ German*. In Berlin *Kiezdeutsch*, this pronunciation variant is rather salient and prevalent in the speech of multi-ethnic youth and their peers from several districts in Berlin. There are differences as to how *Kiezdeutsch* is perceived in the urban population of Berlin: to younger speakers, the multi-ethnic urban variant seems more of a default pronunciation by now and is perceived as *street* (sociolect independent of the ethnic background of the speakers) which stands for young, hip and urban. In contrast, most of the older population and more conservative views published in the press view this sociolect as polarizing, uneducated and negative, and it is shunned upon by mainstream speakers of Berlin German (Heiser, 2014). However, a similar non-canonical like variant also exists in the foreign accented speech of French learners of German which is often seen as cute and endearing and used in TV-advertisements and which generally seems to evoke more positive associations (Giles and Niedzielski, 1998; Plewnia and Rothe, 2009).

In our study we test the implicit attitudes of listener groups varying in age and ethnicity toward identical speech items (a non-canonical pronunciation variant) in the two varying socio-culturally situated contexts of associating these speech forms with multi-ethnic speakers from Kreuzberg and with learners of German from France. Thus, our second hypothesis is, that the implicit association between the non-canonical pronunciation and the negative values is stronger in the *Kiezdeutsch* context than in the *French Learners’ German* context. However, this bias might vary between different listener groups. We assume that social factors such as the age or ethnic background of a listener affects his/her sensitivity to the priming conditions and moreover, his/her attitudes toward the suggested speaker groups. Thus, different biases can be explained in terms of in-group and out-group behavior: the in-group (a cohort that a speaker or listener associates with) is generally evaluated more positively and carries covert prestige compared to the out-group, that a speaker or listener feels socially distanced from Tajfel and Turner (1986) unless the out-group carries high social and cultural prestige. Therefore, the second hypothesis is modified in such a manner that we predict to find our listener groups to vary in the strength of the associations between non-canonical pronunciations and negative values across the priming conditions with regard to their age and ethnic background. Listeners with a multi-ethnic background (similar to the presumed speaker in the *Kiezdeutsch* context) should show a stronger association between non-canonical pronunciations and negative values in the *French Learners German* context (out-group) than in the *Kiezdeutsch* context (in-group). Also, through language experience within the context of urban Berlin, younger (mono-lingual, mono-ethnic German) urban listeners to some degree have overcome their bias toward multi-ethnic and multi-lingual speakers using non-canonical phonetic forms as they themselves perform *street*, resulting in a smaller bias toward non-canonical pronunciations in general independent of the speaker group.

Based on what we have learned from the literature, we presume that hearers notice and recognize fine phonetic detail and index, interpret and evaluate it differentially. We expect our data on the attitudes associated with differentially produced speech features to show that an identical speech variant (a) indexes and receives different social meanings in dependence to the presumed speaker group and (b) receives different social meanings in dependence to the specific hearer group. The study focusses on the saliency of variation in fine phonetic detail in social interpretation and stigmatization of a speaker while exploring the role of the implicit attitudes of different hearer groups interpreting the signal. Thus, it is original and novel insofar as it explores the variance in social meaning of fine phonetic detail in the confines of an urban space in Germany, exploring the role of differences in hearer characteristics (multi-ethnic young; mono-ethnic German and young; mono-ethnic German and older) as well as in- and out-group contextual primes (*Kreuzberg* vs. *French learner of German*).

We set out to test that a phonetic variant is contextualized as it is interpreted in line with usage- and experience-based approaches to language processing (cf. Goldinger, 1998; Barlow and Kemmer, 2000) and therefore, depending on an individual’s experience with and attitude toward speech forms and speaker groups, the same phonetic variant can convey differences in social meaning.

## Materials and Methods

### Acoustic Characteristics of Stimulus Materials

Since the acoustic differences of the auditory stimuli are quite minute yet very crucial, we will provide a short description of the spectral features and acoustic characteristics differentiating the German sibilants /ç/ and /ʃ/ and the pronunciation variant [ɕ]. Although Standard German contrasts three voiceless sibilants phonologically: the alveolar /s/, the postalveolar /ʃ/ and the palatal /ç/, many speakers of the middle German dialects and regiolects do not differentiate between /ç/ and /ʃ/ but use the alveo-palatal pronunciation variant [ɕ] instead. The same holds for multi-ethnic speakers of *Kiezdeutsch* and French learners of German (Wottawa et al., 2016; Jannedy and Weirich, 2017).

Analyses of the acoustic characteristics of different fricatives (Evers et al., 1998; Jongman et al., 2000; Gordon et al., 2002; Nowak, 2006; Cheon and Anderson, 2008; Li et al., 2011), reveal that the acoustic differentiation between /ʃ/, /ɕ/, and /ç/ is rather difficult. For Polish, Czaplicki et al. (2016) found spectral peak (the frequency with the highest amplitude of the spectrum) and center of gravity (CoG, the mean central frequencies for the entire spectrum) to be good predictors to separate a new variant of an alveopalatal fricative from the standard Polish counterparts. Bukmaier and Harrington (2016) investigated the Polish retroflex, dental, and alveopalatal sibilants /ʂ ʃ ɕ/ and while these three fricatives are quite distinct articulatorily, they are difficult to separate acoustically. For German, Jannedy and Weirich (2017) investigated the acoustic difference between /ç/ and /ʃ/ in three speaker groups with varying contrast realizations (one of them being *Kiezdeutsch* speakers from Berlin). While perceptually and acoustically the contrast was lost in the speech of many *Kiezdeutsch* speakers, listeners reliably differentiated these two fricatives in minimal pairs produced by mono-ethnic German speakers from Berlin.

**FIGURE 1:**
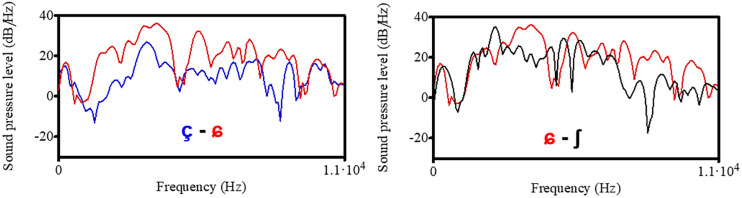
Spectral shapes of the three fricatives [çʃ] produced by a female speaker of Northern German Standard. Different fricatives are marked by different colors.

For a visualization of the acoustic differences in this three-way contrast, Figure 1 shows the spectral shape of [çʃ] produced by a female speaker of Northern Standard German. The two sounds [ç] and [ʃ] correspond to the phonemic fricative categories of Standard German, while [ɕ] is somewhat intermediate between /ç/ and /ʃ/ sounds found in the multi-ethnic urban Berlin variety *Kiezdeutsch* and in French learners’ German. The left plot of Figure 1 visualizes the difference between [ç] (blue) and [ɕ] (red), the right plot shows the difference between [ɕ] (red) and [ʃ] (black). The [ɕ] sound has more energy in the higher frequency range above 5,000 Hz than [ç] and [ʃ]. It also shows a broader band of frequencies with high energy, while both [ç] and [ʃ] reveal clearer peaks but vary in the frequency of this peak: it is higher for [ç] than for [ʃ] which is due to a lengthening of the vocal tract through labialization in [ʃ]. For fricatives, in general, the frequency range with the highest energy is influenced by the place of articulation: the more back the sound is produced, the lower is the CoG. Through the labialization of the alveo-palatal sound /ʃ/ the vocal tract between the lips and the place of constriction is lengthened and thus CoG decreases with [ʃ] having the lowest values. Since the merged sound [ɕ] is produced without lip rounding (in contrast to [ʃ]) and further front than [ç], its spectrum has more energy in higher frequencies and thus has a higher CoG ([ç] = 4,494 Hz, [ɕ] = 5,566 Hz, [ʃ] = 2,575 Hz).

In the experiments conducted in the present study we used stimuli that varied between canonical and non-canonical pronunciations of German, with the non-canonical pronunciation referring to both the *Kiezdeutsch* variety and the French learner variety of German. Therefore, the auditory stimuli used consist of two pronunciation variants of the adjectival suffix <-*ig*> in German, i.e., [ç] for the canonical realization, and [ɕ] for the non-canonical realization.

### Overview of the Study

With a first group of 40 participants, we conducted an online-rating study on the auditory test items to explore if listeners (a) rate the manipulated items to be naturalistic and (b) if and how the pronunciation variations are associated with particular or specific persona types in terms of age or education.

In the main experiment, the IAT, 131 participants were asked to match the presented auditory test stimuli with either a positive or negative valence word. In line with prior IAT results where negative concepts were strongly associated with racial traits that were deemed as less desirable, we hypothesize that non-canonical pronunciations are more strongly associated with negatively connotated words and canonical pronunciations with positive valence words. In general, a non-canonical form is assumed to be evaluated as flawed and bad since it is perceived as deviating from the norm. Thus, overall, reaction times should be faster when the common assumptions are met: when the (negatively connotated) non-canonical pronunciation (categorized as either *Kiezdeutsch* or *French Accent*) and the negative attribute category (i.e., *Bad*) share a response key and when the canonical pronunciation and the positive valence words are mapped to the same response button. A pattern of this type is indicative of an implicit bias of the respondent (Nosek et al., 2005). The robustness of such a mapping is calculated in the form of a single *D*-score per respondent (the greater the bias, the faster the reaction times and the higher the score).

In addition, the particular associations evoked by a non-canonical form are highly dependent on the interpreter, his/her background, stereotypes and beliefs. Thus, the same non-canonical form can be considered as more or less negatively or even positively valenced depending on the attributed prestige of the speaker group associated with the form. Therefore, we hypothesize that the strength of the IAT effect in the two priming conditions *Kiezdeutsch* (condition 1) and *French Accent* (condition 2) is affected by the age and the ethnic background of the listeners due to different biases toward these varieties.

### Online Rating Study

Forty listeners living in Berlin (14 male, 26 female, different from the IAT experiment) were asked in an online rating study to evaluate the naturalness of the stimuli, the supposed level of education and the inferred age of a speaker on a scale from 1 to 7 based on hearing a single word. The experiment was run using *Percy* (Draxler, 2011, 2014). The stimuli tested included the ones used in the IAT experiments and the same words produced by two additional female speakers (each word in the two pronunciation variants). In addition, some filler words were added which are not part of the analysis. The three aspects (*naturalness*, *education*, and *age*) were rated separately in three subsequent blocks and stimuli were randomized over participants. Participants could listen to each stimulus maximally three times. We also collected demographic data of the listeners regarding their age, gender, language background, education, city of birth and current residency as indicated by their postal code.

### IAT Study

#### Participants

In total, 131 German speakers participated in this IAT study, they were distributed into three groups (see Table 1). There were two groups of younger speakers: one was comprised of German born multi-ethnic participants of Turkish or Arab (Lebanese and Palestinian) descent and the second group of younger speakers was comprised of mono-ethnic and mono-lingual Germans. The younger multi-ethnic German listeners (YMU) all were high-school students from Wedding, a multi-ethnic district of Berlin, and stated that they were German language dominant but also often had rudimentary skills in a language other than German. Their German showed several features of the *Kiezdeutsch* variety such as the /ç/- /ʃ/ alternation (Dirim and Auer, 2004; Jannedy and Weirich, 2014), which is neither stigmatized nor recognized amongst them. Younger mono-ethnic German listeners (YMO) were beginning first semester students at Berlin universities who were either born and raised in Berlin or lived there for a significant amount of time of their life. The third group of participants were older mono-ethnic mono-lingual Germans who were born in Berlin or had lived or worked there for over 25 years. We refrained from explicitly asking participants about their familiarity with the concept of *Kiezdeutsch*, since we did not want to prime listeners in any direction. However, in Berlin, the concept of *Kiezdeutsch* is well-known and it can be assumed that everyone living in Berlin for a certain amount of time as our participants have, has at least heard about it in the news, recognizes it when hearing speakers in the tram or even knows someone using it. The same holds for the concept of *French accent* since it is widely used in mainstream media, e.g., in TV advertisement.

**TABLE 1:** Number of participants separated by listener groups with information on ethnic background, gender, and age.

**Listener group**		**No (m/f)**	**Mean age in years (*SD*)**
YMU	**Y**ounger **MU**lti-ethnic German	42 (12/30)	20.67 (4.65)
YMO	**Y**ounger **MO**no-ethnic German	43 (10/33)	25.02 (4.67)
OMO	**O**lder **MO**no-ethnic German	46 (12/34)	51.78 (8.45)

Given these three groups, we were able to compare differences due to ethnic background (mono- vs. multiethnic) and age (younger vs. older) (see Table 1). Participants were randomly distributed into the two different conditions (*Kiezdeutsch* vs. *French Accent*) of the IAT experiment.

#### Methodology and Explanation of Implicit Association Tasks (IAT)

A computer-based IAT requires participants to match stimuli such as orthographically or auditorily presented words with *attribute* or *concept categories* by pressing a button on the keyboard. In our case, words were rendered in the two pronunciation variations *canonical* (=Standard German which we refer to as *Hochdeutsch*) vs. *non-canonical* [=(1) *Kiezdeutsch* (KD, condition 1) or (2) *French Accent* (FR, condition 2)]. It should be noted that the label *Hochdeutsch* which we used in the experiment does not contain the evaluative bias of the word *standard* as in Standard German.

These had to be matched with the *attribute categories* having a psychological valency of either good vs. bad. In other words, pronunciation variants had to be matched to the two language variants (canonical/non-canonical) and words with a positive or negative valence had to be matched to attributes (good/bad).

**FIGURE 2:**
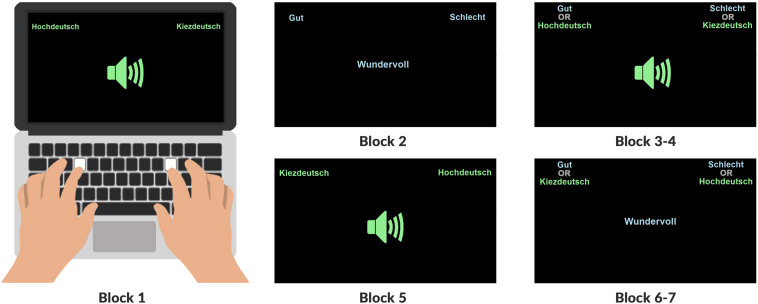
Overview of the order of blocks showing the attribute categories *Gut* (good) and *Schlecht* (bad) in blue and the concept categories *Hochdeutsch* and *Kiezdeutsch* in green in their respective corners. Written and audio stimuli were presented in the middle of the screen.

The trials were divided into seven blocks (see Figure 2 and Table 2): In the first block, participants learn that *Hochdeutsch* pronunciations are mapped to a key (*E*) on the left of the keyboard while non-canonical German pronunciations are mapped to a key (*O*) on the right of the keyboard because the concept category *Hochdeutsch* (cf. Figure 2) appears in the left corner of the computer screen, effectively mapping to the left-hand response key E, and *Kiezdeutsch* (or *French Accent*) appears in the right corner, mapping the right-hand to the response key O. In the second block participants learn that words with a positive valence (good = “gut”, cf. Figure 2) like *“wundervoll”* (*wonderful*) or “*Freude* (*joy*) displayed in the middle of a computer screen, are mapped to the same button on the left side of the keyboard while words with a negative (bad = “schlecht”) valence (like *evil* or *failure*) are mapped to the button on the right side of the keyboard. In the third block, participants are confronted with either a written or an auditory stimulus while simultaneously seeing the label for a concept category like *Hochdeutsch* and an attribute category like *good* mapped to the left button of the keyboard. The label for the concept category *Kiezdeutsch* (or *French Accent*) were mapped to the right button just as the attribute category *bad*. For each sorting operation, the participant’s reaction time is logged. As mentioned above, the hypothesis is that combinations like *canonical variety* + *good* and *non-canonical variety* + *bad* are perceived as congruent and thus generate faster and more immediate reactions in comparison to cases where the implicit bias is violated and non-congruent (*Hochdeutsch* + *bad* and *non-canonical variety* + *good*). In other words, a faster reaction time indicates a stronger association between the paired categories (cf. Nosek et al., 2005).

**TABLE 2:** Overview and order of tasks in the IAT experiments for test order v1.

**Block**	**Trials**	**Task**	**Left key**	**Right key**
1	20	Practice: Audio stimuli only	Concept category *Hochdeutsch* (canonical /ç/)	Concept category *Kiezdeutsch* (or *Franz. Akzent*) (non-canonical /ɕ/)
2	20	Practice: Written stimuli only	Positive valence word *good*	Negative valence word *bad*
3	20	Test: Audio and written stimuli combined	*Hochdeutsch* and *good*	*Kiezdeutsch* (or *Franz. Akzent*) and *bad*
4	40	Test: Audio and written stimuli combined	*Hochdeutsch* and *good*	*Kiezdeutsch* (or *Franz.* Akzent) and *bad*
5	40	Practice reversed: Audio stimuli	*Kiezdeutsch* (or *Franz. Akzent*)	*Hochdeutsch*
6	20	Test reversed: Audio and written stimuli combined	*Kiezdeutsch* (or *Franz. Akzent*) and *good*	*Hochdeutsch* and *bad*
7	40	Test reversed: Audio and written stimuli combined	*Kiezdeutsch* (or *Franz. Akzent*) and *good*	*Hochdeutsch* and *bad*

**For v2, the concept categories Kiezdeutsch/Franz. Akzent and Hochdeutsch switched keys resulting in incongruent test blocks first (3 and 4) and congruent test blocks second (6 and 7)*.*

In the third and also in the fourth block, all four categories appeared combined, pairing a concept category and an attribute category with one response key each. These two blocks constitute the congruent test cases (according to our hypothesis) and contain one half of the experimental trials from which the final IAT effect is calculated. The fifth block is a practice block for audio stimuli again, which introduces a crucial manipulation: while the attribute category mapping is kept constant throughout the experiment, the concept category mapping learned in the previous blocks is inverted by switching the position of the concept category labels (e.g., *Kiezdeutsch/French Accent* now maps to the left key and *Hochdeutsch* maps to the right key), effectively leading to an incongruent (according to our hypothesis) and therefore supposedly more difficult matching task. The number of trials in the fifth block is increased (40 instead of 20 trials in the practice blocks 1 and 2 before) to compensate for the learned mapping reinforced by all preceding trials (Nosek et al., 2005).

Blocks 6 and 7 combined all four category labels again while maintaining the incongruent category labels from block 5. These final two blocks provide the other half of the experimental trials needed for calculating the IAT effect size (called *D-score*). For each participant in the study, a single *D*-score value is calculated. *D*-scores are computed as the mean difference between test blocks divided by the overall standard deviation of latencies. A detailed account of the scoring algorithm can be found in Greenwald et al. (2003). A *D*-score close to zero means no IAT effect at all. A positive *D*-score reveals a closer association between non-canonical pronunciations and “bad” and canonical pronunciations and “good” (in line with our hypothesis), while a negative *D*-score shows a closer association between canonical pronunciations and “bad” and non-canonical and “good” (opposed to our hypothesis).

In order to avoid block order effects, the starting position of the concept category labels was counterbalanced across participants. Thus, the order of test blocks – congruent to our hypothesis (*Hochdeutsch* + *good* and *non-canonical German* + *bad*) and incongruent with our hypothesis (*non-canonical German* + *good* and *Hochdeutsch* + *bad*) – was varied between participants. Half of the participants did the congruent test blocks first (as described above), the other half did the incongruent test blocks first, leading to two versions of the experiments (v1 and v2, cf. Table 2). Participants were randomly assigned to the different experiment conditions (KD and FR) and order versions (v1 and v2) resulting in 10–12 participants in each of different listener groups (YMO, YMU, and OMO). Note that for participants in the FR condition all instances of *Kiezdeutsch* were replaced with *French Accent.*

The experiment was run on a Lenovo IdeaPad U330 laptop with 1,366 × 768 screen resolution using PsychoPy2 v1.85.3 (Peirce and MacAskill, 2018). For presentation of the auditory stimuli, Sennheiser HD590 headphones were used. The order of presentation for auditory and visual stimuli was randomized for each block across all participants. Overall, the experiment took approximately 20–30 min per participant including a questionnaire about some metadata of the participants.

#### Materials

The viability of using auditory stimuli in an IAT paradigm was first shown by Vande Kamp (2002) and has since been used in a variety of linguistic studies (Pantos, 2010; Campbell-Kibler, 2012, 2013; Pantos and Perkins, 2012; Loudermilk, 2015; Hilton et al., 2016; Leinonen, 2016; Llamas et al., 2016; Rosseel et al., 2018). For the current study, a female native mono-ethnic German speaker from Berlin (age 27) read 6 German adjectives ending in the syllable <*-ig*>: *einzig* “solely,” *fertig* “ready,” *mehlig* “floury,” *nussig* “nutty,” *körnig* “grainy,” *bündig* “concisely” in two different versions with two different pronunciation variants: *Hochdeutsch* and *Kiezdeutsch* (condition 1), doubling up as French learners’ accent in German (condition 2). As described above, in *Hochdeutsch*, the final sound is pronounced as a voiceless palatal fricative [ç], while it is pronounced as [ɕ] in *Kiezdeutsch* and in French learner varieties of German. These 12 recordings were used as the auditory stimuli for the IAT. Recordings were made in a sound attenuated room with a head-mounted Sennheiser MKH 50 P48 microphone at 44 KHz. Recordings were downsampled to 22 KHz for use in the study.

**FIGURE 3:**
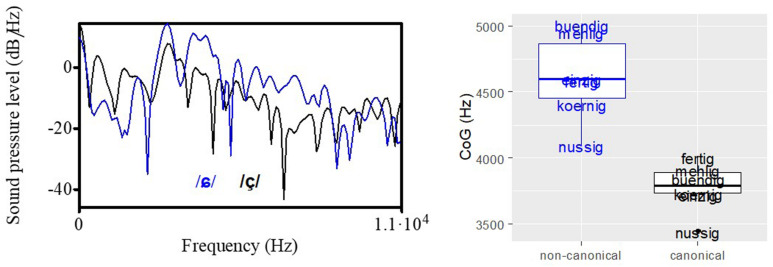
**(Left plot)** Spectral shape of the two fricatives in /nʊ.sɪç/ (black) and /nʊ.sɪɕ/ (blue). **(Right plot)** Distribution of CoG values separated by canonical (black) and non- canonical (blue) stimuli.

Figure 3 (left plot) shows the spectral shape of the two fricatives in /nʊ.sɪç/ (black) and /nʊ.sɪɕ/ (blue). The shift to the higher frequencies for the non-canonical /ɕ/ pronunciation (probably due to a more fronted articulation) can be seen and mirrors the acoustic description of the different sibilants in Section “Acoustic Characteristics of Stimulus Materials.” The greater energy in the higher frequencies can be captured by CoG values, shown in the right plot of Figure 3. The fricatives of all word pairs are characterized by a difference in CoG, with higher values for the alveo-palatal fricative /ɕ/ as realized in the non-canonical variety.

**FIGURE 4:**
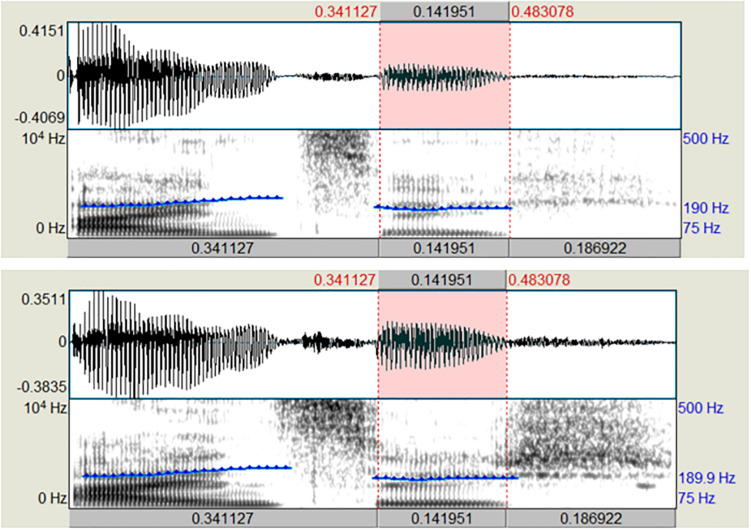
Spectrogram and oscillogram of the *einzig*-pair with temporally adjusted segments and normalized pitch contour (above: canonical realization [ʔaɪntsɪɕ], below: non-canonical realization [ʔaɪntsɪç]).

To use the auditory stimuli in the IAT experiment, they were temporally normalized. To do so, the stimuli were segmented into three parts: stem + /I/ + /ç/. Each part was manipulated to have a certain length (0.34, 0.14, and 0.19 s, respectively). This was done, so that the [ɪç] part of each stimulus word had the same duration across all stimuli and the duration of the stem was kept constant. In addition, the stimuli were normalized in amplitude (mean intensity of 70 dB) and fundamental frequency (f0). The normalization of f0 was done in a pairwise fashion by synthesizing the non-canonical rendition of a word pair with the extracted f0 contour of the canonical stimulus of the same word pair. This was done to control for differences between *Hochdeutsch* vs. the two non-canonical conditions but at the same time keeping the stimuli as natural as possible. Mean f0 varied between the word pairs from 204 Hz for the *einzig*-pair to 214 Hz for the *bündig*-pair. All manipulations were carried out using *Praat* (Boersma and Weenink, 2018). Figure 4 shows spectrogram and oscillogram of the *einzig*-pair with temporally adjusted segments and normalized pitch contour (above: canonical realization, below: non-canonical realization). All test items were rated for their naturalness prior to using them in the IAT experiment (see Result section “Online Rating Study”).

Stimuli for the attribute categories consisted of 12 visually presented lexical items with either a positive or a negative valency. We selected these 12 words from a range of items suggested on the German sample IAT site “Project Implicit” hosted by Harvard University^1^. The words with a positive valency were: *Freude* “joy,” *Frieden* “peace,” *Lachen* “laughter,” *Liebe* “love,” *Vergnügen* “pleasure,” *wundervoll* “wonderful.” The negatively connotated words were: *böse* “evil,” *grausam* “cruel,” *Misserfolg* “failure,” *Qual* “agony,” *Übel* “evil,” *verletzt* “hurt.” These attributes were selected because of their frequent and prior use in previous IAT studies.

We ran two versions of this experiment: in condition 1, half of the participants saw the opposing concept categories *Hochdeutsch* and *Kiezdeutsch* while in condition 2, the second half of the participants saw the opposing categories *Hochdeutsch* and *French Accent*. Both versions of the experiment differed in the introductory text shown on the screen. In condition 1, called KD-experiment below, 68 participants (22 YMU, 20 YMO, and 26 OMO) were informed in the introduction that the auditory stimuli were recordings obtained from students at a school in the multi-ethnic district of Kreuzberg in Berlin. Accordingly, the concept categories for sorting the auditory items were labeled *Hochdeutsch* and *Kiezdeutsch*. In condition 2, called FR-experiment below, 63 participants (20 YMU, 23 YMO, and 20 OMO) read in the introduction to the experiment that they were listening to recordings of French students learning German. The concept category labels were *Hochdeutsch* and *Franz. Akzent* (*French Accent*). Both groups were told that the aim of the experiment was to sort the presented stimuli (both auditory and written) correctly into the given categories and that it was important to do this as fast as possible.

#### Hypotheses

Since the stimuli were the same across both versions of the experiment and for all participants, a difference in judgments reveals whether the same phonetic alternation is judged differently by each participant group depending on the information received on the origin of the auditory stimuli. As mentioned above the following hypotheses are tested:

(1) Canonical pronunciations are associated with positive values, non-canonical pronunciations with negative values (IAT-effect).
(2) The socially situated context (French Lerner German vs. *Kiezdeutsch*) biases listeners to interpret the identical acoustic stimuli differentially depending on the listeners’ age and ethnic background.

(a) Mono-ethnic German listeners show a stronger IAT-effect in the KD experiment indicating a stronger negative bias toward this variety than in the FR experiment, while multi-ethnic listeners show a reversed pattern with a stronger negative bias toward the supposed French variety than toward their own speech group.
(b) Younger mono-ethnic German listeners show a smaller IAT-effect than older mono-ethnic German listeners mirroring their smaller bias toward non-canonical pronunciations.

#### Statistical Analysis

Statistical analyses were conducted in *R* (R Core Team, 2016). We ran one-sample t-tests and linear (mixed) models using the packages *lme4* (Bates et al., 2015) and *lsmeans* (Lenth, 2016). Significance testing was done by model comparison (with and without the factor or interaction in question). For the online rating test, the fixed factors included were *word*, *pronunciation variety* and *speaker*, while *listener* was added as random effect. For the IAT analysis, just one *D*-score per participant (across words) constitutes the dependent variable, thus, the calculated linear models include *listener group* and *test order* (version) as predictors, and additionally *experiment condition* in the combined data set.

## Results

### Online Rating Study

**FIGURE 5:**
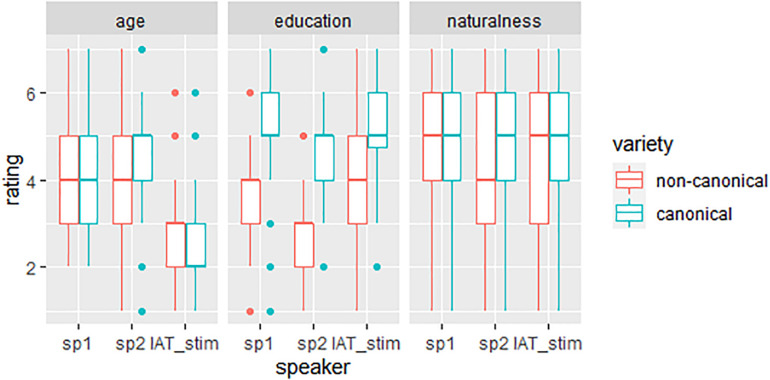
Distribution of ratings regarding perceived age, education and naturalness separated by speaker (sp1, sp2, and IAT_stim) and variety (blue = /ç, red = /ɕ).

Figure 5 (right panel) shows the results of the online rating study with 40 participants, that tested for the perceived naturalness of the auditory IAT stimuli (named *IAT_stim*) in comparison to non-manipulated items produced by two different female speakers (*sp1*, 39 years and *sp2*, 53 years). In addition to the perceived naturalness, we tested the perceived age and education of the speakers, shown in the left and middle panel of the figure. Ratings are separated by pronunciation variation (blue: canonical = /ç/, red: non-canonical = /ɕ/).

Most importantly, the manipulation did *not* show an effect on perceived naturalness: the manipulated stimuli used in the IAT did not differ significantly from the other stimuli. However, the LMM showed a main effect of word [χ^2^(5) = 18.42, *p* < 0.01], with *bündig* and *einzig* being perceived as less natural. A possible explanation is that these words are less frequent in their use than the other adjectives, especially when used out of context. More interestingly, we found an interaction of speaker and pronunciation variation [χ^2^(2) = 12.01, *p* < 0.01]: ratings were significantly less natural for the non-canonical pronunciations than for the canonical pronunciations for two out of the three speakers (sp2 and the IAT_stim, *p* < 0.001), but not for sp1. This might reflect the fact that sp1 is a native speaker of a central German dialect who produces the versions of the <*-ig>* with a less salient perceptual contrast, whereas the other two speakers differentiated more clearly between a canonical and a non-canonical pronunciation.

For age, we also found an interaction of speaker and pronunciation variation [χ^2^(3) = 43.32, *p* < 0.001]: Sp1 again did not show a difference in perceived age between the pronunciations. While sp2 sounded *older* in canonical than non-canonical, the IAT speaker sounded *younger* in canonical than non-canonical. This opposing effect of pronunciation on perceived age in the two speakers is striking at first but might be due to the difference in biological age between the two speakers. For a young woman in her 20s a non-canonical pronunciation increases the perceived age, while it decreases the perceived age in a woman in her early 50s. For education, the interaction of word, pronunciation variation and speaker turned out significant [χ^2^(22) = 92.09, *p* < 0.001]. While there was variation in terms of differences between speakers (in some words and a certain pronunciation), for all speakers and all words the non-canonical variation was perceived as less educated than the canonical pronunciation (cf. Figure 5).

### Error Rates and Reaction Times of the IAT Studies

#### Error Rates

As a first step to the analysis of the data obtained in the IAT experiment, we performed an error analysis to check whether participants were able to discriminate between the two pronunciation variants above chance level and to compare the correctness scores to the ones for the written stimuli. Overall, correctness scores were high, but as expected, they were higher in the written stimuli than in the audio stimuli. In the practice trials (Block 1: audio only, Block 2 written only), participants identified the audio stimuli in 84% correctly, the written stimuli in 98%. Table 3 shows the correctness scores for the identification tasks separated by participant group and stimulus type (audio vs. written) with all blocks included. Again, the numbers show that the written stimuli were more easily correctly identified by the participants than the auditory stimuli. There were no obvious differences between the context conditions (FR vs. KD) in correctness scores, however, there is a slight tendency for the audio stimuli to be identified more reliably in the KD condition compared to the FR condition. However, this is only true for the monolingual groups YMO and OMO, but not for the YMU listeners, who do not differ between conditions and overall displayed the poorest performance in identifying the audio stimuli.

**TABLE 3:** Average correctness scores (in %) of audio and written stimuli calculated over the whole experiment separated by participant group and context condition (French Accent vs. Kiezdeutsch).

**Participant group**	**FR (audio/written)**	**KD (audio/written)**
YMU	80.2/92.1	79.8/95.4
YMO	88.4/95.9	93.1/95.8
OMO	86.7/96.1	91.9/96.3

#### Reaction Times

Here, a short description of participants’ absolute reaction times (RT) is given. Without practice trials and independent of stimulus type (audio/written) and experiment (KD/FR), RT was on average 1,144 ms (measured from the time when stimuli were displayed on screen or played) and ranged between a lower and upper quartile of 764 and 1,299 ms. Table 4 shows the reaction times separated by experiment, stimulus type and shared key conditions. Overall, participants in the KD experiment were slightly slower than in the FR experiment across all subgroups. Note though, that these overall differences between experiment conditions do not affect the IAT effect (*D*-score), since this measure is calculated for each participant separately and is a comparative measure which takes the relation of the RTs of the different blocks into account. Also, in general, written stimuli were categorized faster than audio stimuli. However, it should be kept in mind that the audio stimuli had a length of 670 ms and differed only in the final sound, i.e., the canonical or non-canonical fricative, and thus reducing the time participants took to decide. Most interestingly, it took participants less time to sort stimuli into categories when these categories were placed congruent to our hypotheses (“canonical form” and “good” sharing the same key) across both stimulus types and experiments.

**TABLE 4:** Participants’ mean reaction times (RT) and standard deviations (SD) separated by experiment condition (KD and FR), shared keys (congruent and incongruent to our hypothesis) and stimulus type (audio and written).

**Experiment**	**Shared keys**	**Mean RT in ms (*SD)* audio stimuli/written stimuli**
KD	Non-canonical and good	1475.9 (820.9)/1180.6 (758.0)
	Canonical and good	1196.1 (710.4)/913.0 (562.2)
FR	Non-canonical and good	1366.3 (677.4)/1063.9 (688.0)
	Canonical and good	1124.7 (452.6)/833.6 (505.7)

### IAT Condition 1: KD-Experiment

**FIGURE 6:**
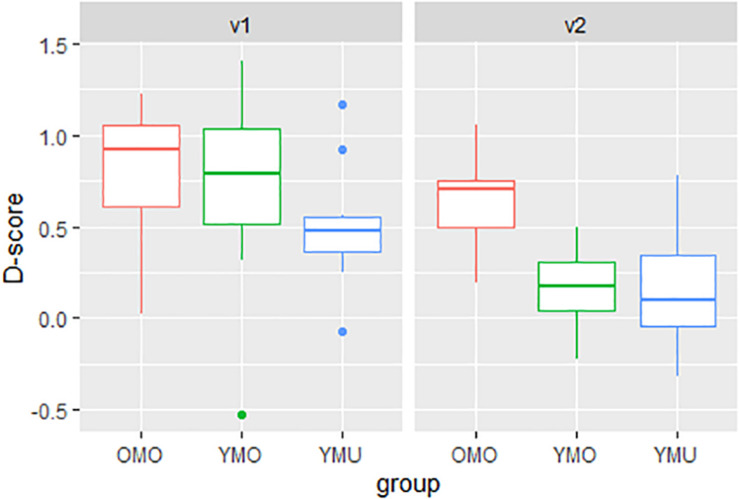
Distributions of *D*-scores (pronunciation effect, *y*-axis) as a function of listener group (YMU, YMO, and OMO) and order (test version v1, v2) for the KD-experiment.

Figure 6 shows the distribution of *D*-scores separated by listener group (YMU, YMO, and OMO) and order of presentation (v1 and v2). As mentioned above, in v1 participants did the congruent pairing first (canonical – positive, non-canonical – negative), in v2, participants started with the incongruent pairing (non-canonical – positive, canonical – negative). Remember that a *D*-score near zero indicates that there is no effect of the experiment. A positive *D*-score, however, means analogous to our hypothesis that the non-canonical /ɕ/-pronunciation was associated with negative adjectives and the canonical /ç/-pronunciation was associated with positive adjectives. The figure shows a clear difference between the test orders (v1 vs. v2), it matters which pairing was seen and learned first. Test version v1 seems to generally result in a stronger IAT effect (higher and more positive *D*-scores) than version v2. Differences are also apparent between the listener groups. The oldest group OMO reveals the highest *D*-scores (most strongly associating the non-canonical pronunciation with negative valence words) and group YMU the lowest, while group YMO lies in between the two.

One-sample *t*-tests were carried out across v1 and v2 for each listener group separately to see whether the *D*-scores deviate from zero thereby indicating a positive IAT effect. Significant effects were found for all listener groups corroborating hypothesis 1 [YMU: *t*(19) = 4.01, *p*-value < 0.001, YMO: *t*(19) = 3.935, *p*-value < 0.001, OMO: *t* = 11.261 (21), *p*-value < 0.0001]. To investigate more closely the size of the IAT effect depending on the order of presentation and the listener group, a linear model was calculated with the *D*-score as dependent variable and the factors listener group (YMU, YMO, and OMO) and order of presentation of the test items (v1 and v2) as the predictors. Table 5 shows a summary of the results. As indicated already in Figure 6, a significant ordering effect was found independently of listener group (even though the effect seems to be strongest for the YMO group – green in Figure 6 – the interaction was not significant): the IAT effect was larger, meaning there was a stronger association between the non-canonical pronunciation /ɕ/ and the negative adjectives in version 1 (v1) where experiment participants first practiced the association of the /ɕ/ pronunciation with the negative adjectives.

**TABLE 5:** Summary statistics of the linear model with *D*-score as dependent variable and the influencing factors *listener group* and *test order* for the KD-experiment.

	**Estimate**	**Std. error**	***t*-value**	**Pr(>| *t*|)**
(Intercept)	0.25337	0.09059	2.797	0.006991**
Group YMO vs. YMU	−0.09315	0.11140	−0.836	0.406512
Group YMO vs. OMO	0.30858	0.10884	2.835	0.006295**
Group YMU vs. OMO	0.40173	0.1088	3.691	0.000495***
Order v2 vs. v1	0.34662	0.08948	3.874	0.000275***

^∗∗^*p* < 0.01, ^***^p < 0.001.

More interestingly, a significant effect of listener group was found with the older listeners (group OMO) differing from both the younger multi-ethnic (YMU) and younger mono-ethnic (YMO) listeners, while the difference between YMO and YMU does not differ significantly. Thus, the IAT effect regarding the association between negative adjectives and the non-canonical /ɕ/-pronunciation was larger for the older listener group compared to the younger groups corroborating hypothesis 2.

### IAT Condition 2: FR-Experiment

Parallel to the analysis of the KD-experiment one sample t-tests were made for each listener group to see whether *D*-scores differ significantly from zero and thus indicate a positive IAT-effect. As in the KD-experiment, significant IAT-effects were found for all listener groups [YMU: *t*(19) = 8.5608, *p*-value < 0.0001, YMO: *t*(22) = 4.1255, *p*-value < 0.0001, OMO: *t*(19) = 6.9851, *p*-value < 0.0001].

**FIGURE 7:**
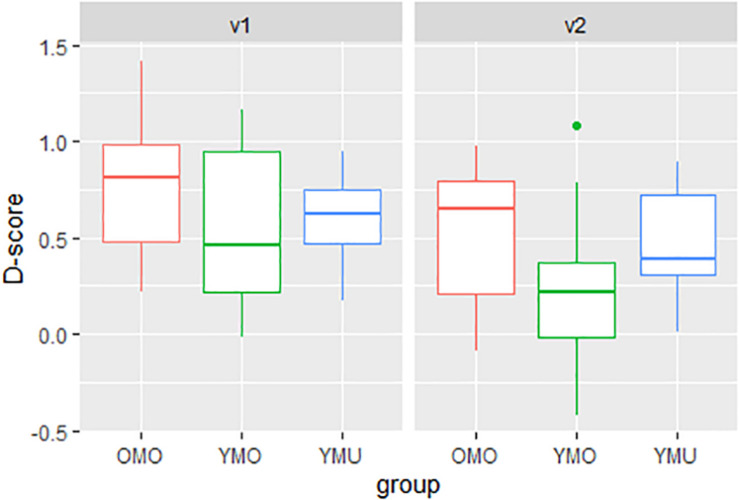
Distributions of *D*-scores (pronunciation effect, *y*-axis) as a function of listener group (YMU, YMO, and OMO) and order (test version v1, v2) for the FR-experiment.

Figure 7 shows the distribution of *D*-scores separated by listener group and order of presentation. Similar to the KD-experiment, variation between listener groups and versions appear to be pointing to listener- and order-specific differences in the size of the IAT-effect. However, in comparison to the KD-experiment, the intra-group variability is much greater when experiment participants believed to be listening to French learners of German. This is reflected by the larger box sizes comprising 50% of the data above and below the bold line (median) in each bar. Also, here, group YMO shows the lowest mean *D*-score whereas in the KD-experiment, YMU displayed the lowest score.

To test for the significant differences between listener groups and test orders, here too, a linear model was calculated. Again, significant main effects of the two factors *group* and *order* were found but no interaction thereof. Table 6 shows the summary statistics of the model with a stronger IAT effect in version 1 than in version 2 and a stronger IAT effect in group OMO compared to YMO. While the *D*-scores of group YMU lie in between the other two groups, the differences fail to reach significance which we assume is also affected by the large intra-group variation that can be seen in Figure 7.

**TABLE 6:** Summary statistics of the linear model with *D*-score as dependent variable and the influencing factors *listener group* and *test order* for the FR-experiment.

	**Estimate**	**Std. error**	***t*-value**	**Pr(>|*t*|)**
(Intercept)	0.26598	0.08788	3.027	0.00366**
Group YMO vs. YMU	0.14271	0.11157	1.279	0.20588
Group YMO vs. OMO	0.23088	0.11157	2.069	0.04290*
Group YMU vs. OMO	0.08818	0.11538	0.764	0.4478
Version 2 vs. 1	0.24347	0.09197	2.647	0.01039*

*^∗^*p* < 0.05, ^∗∗^*p* < 0.01.*

### Comparing the Experiments KD and FR

**FIGURE 8:**
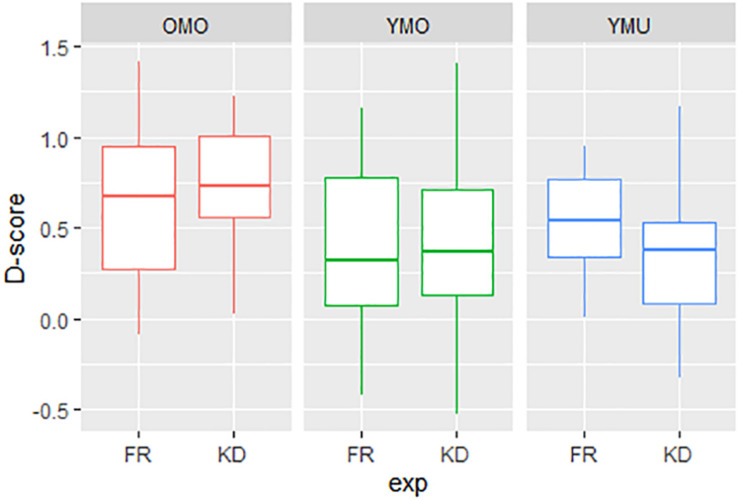
Distributions of *D*-scores (pronunciation effect, *y*-axis) as a function of listener group (YMU, YMO, and OMO) and experiment (KB and FR).

We will now take a closer look at the similarities and differences between the two test conditions (KD and FR) by combining the data sets. Figure 8 gives a first impression, showing the *D*-scores for both order versions separated by listener group (YMU, YMO, and OMO) and experiment (FR and KD). Values for the younger mono-ethnic hearers YMO (green in Figure 8) are in between the two other groups, and more importantly, there is no obvious difference between the two conditions FR and KD. For the older listeners (red) and the younger multi-lingual listeners (blue), the effects go in different directions: while for group YMU the FR experiment reveals higher *D*-scores and thus a stronger IAT effect, for group OMO the KD experiment reveals slightly higher *D*-scores. The YMO group seems widely unaffected by the different priming conditions, the *D*-scores are above zero but rather low (with a large spread indicative of the variance in the responses) for both the KD and FR condition.

A linear model was calculated over the combined data with *D*-score as dependent variable and the factors order (v1 and v2), listener group (YMU, YMO, and OMO) and condition (KD and FR) as potential influencing variables. Table 7 shows the summary statistics of the model. This time, in addition to the main effect of test order, a significant interaction between listener group and condition was found as suggested by hypothesis 2. As already indicated in Figure 8, the difference in the IAT-effect between the experiment conditions varies between OMO and YMU: While for the YMU-listeners there was a larger IAT-effect in the FR condition, reflected by the median *D*-score in the Figure and a negative estimate in Table 7 (line 2), for OMO-listeners, the larger IAT-effect was found in the KD-condition reflected in the significant interaction and a positive estimate in Table 7 (last line).

**TABLE 7:** Summary statistics of the linear model with *D*-score as dependent variable and the influencing factors *listener group*, *test order* and experiment *condition*.

	**Estimate**	**Std. error**	***t*-value**	**Pr(>|*t*|)**
(Intercept)	0.38310	0.08625	4.441	2.03e-05***
Condition KD vs. FR (for YMU)	−0.1968	0.11325	−1.738	0.0847
Group OMO vs. YMO	−0.1415	0.10951	−1.293	0.1985
Group OMO vs. YMU	0.08818	0.11325	0.779	0.4378
Version 2 vs. 1	0.29465	0.06408	4.59	1.08e-05***
Condition KD * Group YMO	0.23474	0.15754	1.490	0.1389
Condition KD * Group OMO	0.31356	0.15834	1.980	0.0500*

^∗^*p* < 0.05, ^***^p < 0.001.

This reveals that for older listeners the association between the /ɕ/ pronunciation and negative valency words was stronger when linked to the *Kiezdeutsch* variety, for the YMU group the association between the non-canonical variant and negative valency words was stronger when linked to a French learner variety than to *Kiezdeutsch* – the variety that many of the listeners themselves speak and are habituated to. YMO listeners lie between the other groups with no obvious difference in bias between the two cultural priming contexts.

## Summary and Discussion

Our results indicate that the experimental paradigm was successfully deployed to show that the implicit attitudes of the three different hearer groups not only differ but also, that the two different contexts elicited differences in implicit associations. There is a priming effect of the variant fricative forms that carry social meanings and trigger implicit attitudes. Based on the speed of reaction to the stimuli, we argue that the patterns observed here are unmediated and indicative of implicit attitudes. The method that we have chosen in our study has shown repeatedly that individuals process information implicitly (i.e., automatically or unconsciously) (Greenwald and Banaji, 1995; Bargh and Chartrand, 1999; Banaji, 2001; Greenwald et al., 2002, 2009; Fatfouta et al., 2014) as opposed to explicitly (controlled or conscious). To be more precise, we argue that our results show attitudes below the level of consciousness due to three different aspects related to the IAT method.

First, the reaction times of the responses range between a lower and upper quartile of 764 and 1,299 ms, which points to a rather rapid overall response. As a reference, in neural language processing EEG (electroencephalography) studies, a negative deflection in the ERP (event related potentials) signal at around 400–550 ms (N400) after stimulus onset indicates a detection of semantic anomalies (see for example Van Berkum et al., 2008). Given that these are very immediate and pre-motor brain responses that do not require any decision making (left or right button) or activation of motor patterns (such as lifting a finger and pressing a button), the average RT in our IAT-study seems relatively fast.

Second, participants are generally not aware of what is being measured in an IAT experiment. They might of course notice having more trouble when *good* and *Kiezdeutsch* are mapped to the same response key. However, participants might not attribute this directly to a bias they have. On the contrary, participants are often negatively surprised by their results showing a bias against a specific group of people as they are generally not aware of it (see Banaji and Greenwald, 2013). So far, it is unclear if implicit biases are based on, e.g., personal experiences, learned mainstream attitudes based on frequent confrontation with stereotypes, or internalized stereotypes against out-groups or even the own in-group.

Third, even if participants are aware of the scoring algorithm, they are unlikely able to consciously alter and adjust their behavior with the purpose of influencing their final score. For example, in case of trying to hide a bias against the *Kiezdeutsch* variant, one would have to deliberately take longer in the blocks congruent with our hypothesis (*bad* – *Kiezdeutsch* and *good* – *Hochdeutsch*) while also trying to be faster in blocks incongruent to our assumption (good – *Kiezdeutsch* and *bad* – *Hochdeutsch*). Moreover, this strategy would have had to have been maintained throughout the entire experiment which would have added to the cognitive load and would inevitably have led to an overall increase in RT. And as a last point, there is a systematic pattern of variation within but not across the three listener groups, showing that all of them have different implicit associations with the stimuli presented.

That is, we see that not all groups behave alike. Our first hypothesis that non-canonical pronunciations are more strongly associated with negatively valence words and canonical pronunciations with positive valence words is borne out. This finding is also corroborated by the results of the online rating experiment where stimuli from all three speakers and all words are rated less educated in the non-canonical pronunciation variant than in the canonical pronunciation. In addition, the distribution of absolute reaction times mirrors the results of the IAT experiments: participants were faster in sorting the audio and written stimuli into the corresponding categories when these categories were placed according to our hypothesis (canonical pronunciations and positive valence words, and non-canonical pronunciations and negative valence words sharing the same response keys).

Moreover, in accordance with our second hypothesis, we could show that the IAT effect differs between listener groups: it is stronger for the older age group compared to the younger groups regardless of their language and ethnic background. In addition, for the older listener group we find a greater effect (a stronger association of non-canonical pronunciation and negative valence words) when hearers believe to be listening to a speaker from Kreuzberg and a lesser effect (indicating a less negative attitude toward this speaker group and speech variant) when hearers believe to be listening to a learner of German from France. While the younger mono-ethnic German group seems indifferent toward the priming conditions (FR vs. KD), the younger multi-ethnic group links the variant pronunciation [ɕ] more strongly with negative valence words in the French learner condition compared to the *Kiezdeutsch* condition, showing a preference for their in-group.

It therefore seems that non-canonical speech forms must not necessarily evoke negative associations and are highly dependent on the interpreter (covert prestige). This is a complex and evolving process especially considering the ongoing diversification of the urban Berlin population but also many other urban spaces in Europe [e.g., Multicultural London English, Kerswill et al., 2008; Straattaal (Netherlands), Nortier, 2001; Rinkeby-Svenska (Sweden), Kotsinas, 1998; Kobenhavnsk Multietnolekt (Denmark), Quist, 2005; Multiethnolektales Schweizerdeutsch (Switzerland), Schmid, 2020; Kebab Norsk (Norway), Svendsen and Röyneland, 2008] where there are many antagonistic but also collective forces that build a microcosmos of their own and where world knowledge may be shared but differently evaluated, categorized or interpreted. The concepts of ethnolectal group membership (in-groups vs. out-groups, cf. Tajfel and Turner, 1986) are categories that are somewhat augmented by social affiliations with aspects of mainstream and non-mainstream culture.

Berlin prides itself with being an open-minded, diverse, friendly and multi-cultural European city with a truly international flair due to the ethnic diversity of its inhabitants and the many tourists. Especially younger people from Berlin embrace this urban feel and the flair of the hip and diverse neighborhoods. As such, there is some cultural capital (Bourdieu, 1986; Jannedy et al., 2019) associated with having international affiliations, being of multi-ethnic decent and well versed in *street*-culture. It is not that the speech features described for *Kiezdeutsch* are intrinsically hip or cool – in fact, there is some evidence that the mainstream is not fond of the linguistic variation – it is the hipness of the concept of being part of the underdog, bad-boy, *street*, and youth-culture, of being shunned upon by more conservative forces and by integrating with those who in the past were not well integrated by embracing aspects of their culture, food, style, and speech. In other words, speech features that were associated with one specific social group (i.e., multi-ethnic adolescents) and that are stigmatized especially by conservative forces, were used by other parts of the younger urban population through *crossing* (Dirim and Auer, 2004, pp. 204–224; Rampton, 2014), have gained covert prestige, and were adopted as their own, indexing social orientation toward multi-ethnicity, diversity, and urbanity.

In light of this, it is feasible that younger listeners in general are more open to variation in fine phonetic detail as they are in a better position to contextualize phonetic innovations and accept these as potentially meaningful expressions of identity while older populations are more strongly attached to a fictitious standard. Our work on the /ç/ – /ʃ/ merger in Berlin (Jannedy and Weirich, 2014) corroborates these assumptions as identification patterns of older listeners showed more [ɕ] ratings when they believed that the speaker was from a multi-ethnic district (i.e., *Kreuzberg*) compared to a mono-ethnic German district of Berlin, while younger listeners were not receptive to the priming. Especially for the older listeners, the data strongly indicates that there is a lack of social status and prestige associated with the pronunciation of /ç/ as [ɕ] when attributed to a speaker group from *Kreuzberg*. The IAT results also corroborate the finding that younger mono-ethnic listeners seem to have less of a strong bias toward one variant over another with *D*-scores only slightly above zero in both conditions. We suspect this being due to hearing both versions in the ambient environment and maybe even variably producing it in contexts that situationally or functionally demand not using a canonical version of this fricative.

The interesting effect of age reflected in the results indicates that the oldest group of listeners (OMO) had the strongest associations of the non-canonical variant [ɕ] with negative valence words in both conditions, with a slightly stronger tendency in the *Kiezdeutsch* condition. Thus, a phonetic variant stemming from a French learner variety of German did (even though only to some extent) evoke more positive associations than the multi-ethnic variant associated with *Kiezdeutsch*. The results for the oldest listener group and the younger multi-ethnic listener group are diametrically opposed: listeners in the YMU group had stronger negative associations with the French variety compared to the *Kiezdeutsch* variety. Not only is this evidence that the associative responses are learned but also that in the case of the YMU group, the responses toward the in-group variety *Kiezdeutsch* that many of the listeners themselves speak and are habituated to were more positive (cf. Tajfel and Turner, 1986). In addition, the analysis of the error rates revealed that this group had the most problems in differentiating the two pronunciation variants, probably reflecting their own productions of the merged variant typical for this speech community and also, a lesser awareness of the distinction in general.

For each experimental condition (KD/FR), we have tested a different group of hearers to prevent having to draw attention to the contrast between these conditions which would have biased hearers in an uncontrollable manner. To prevent this from happening and of course also due to time constraints and participants fatigue we assigned participants randomly to two groups differing in the experiment conditions. However, in order to investigate the effect of priming condition (French accent vs. *Kiezdeutsch*), we collapsed the data for these two different conditions. The important point is that we found an interaction between *listener group* and *condition* (for some groups the prime *Kiezdeutsch* evokes stronger IAT effects, for others the prime *French Accent* did so). We therefore assume that these results are due to different associations drawn by the different listener groups and not due to different participants taking part in the two experimental conditions (because then we would have expected a main effect of experiment, with an overall difference between conditions irrespective of listener group).

In addition to the hypothesized effects of priming condition and listener group, our results also show an effect of which associations listeners rated first. IAT effects were generally larger when the version with the hypothesis-congruent relations were shown first (canonical pronunciation and positive value sharing a response key) followed by the hypothesis-incongruent relations (canonical pronunciation and negative value). These order effects (i.e., associations appear stronger when they are tested in blocks 3 and 4 rather than in blocks 6 and 7) have been described earlier and a suggested improvement of IAT experiments was to increase the number of trials in block 5 to counteract these order effects (cf. Greenwald et al., 2003). In our study however, order effects still appeared despite this change in method. Greenwald et al. (2003, p. 209) suggest that order effects in IATs might be related to a phenomenon called *negative transfer* (Woodworth et al., 1954), “whereby practice at one task interferes with performance at a second task that requires giving different responses to the first task’s stimuli.” This negative transfer is assumed to result in a strengthening of associations between the hypothesis congruent categories (canonical-good) when the task that uses this association (that uses the same response keys to canonical pronunciations and positive words) is tested initially.

With respect to our study it seems that the IAT effect is somewhat leveled when listeners first “learn” the incongruent association and must redo this learning with a new (but) better fitting association (in terms of their implicit bias). When the listeners first “learn” the congruent association according to their implicit bias, the bias is strengthened and the redoing of the learned (and fitting to their stereotypical) association is even more difficult resulting in a stronger IAT effect. However, this was the case in both priming conditions (*French accent* and *Kiezdeutsch*) and no interaction of order (version) and priming was found. Also, there was no significant difference between the listener groups in terms of the order effect, even though a tendency was apparent for the YMO group in the KD experiment to show a larger difference between the versions than the other listener groups (mainly due to a very small *D*-score and thus a low IAT effect in version 2). Thus, the YMO group shows the strongest effect of test order which might point to a greater flexibility in their associations between pronunciation variants (canonical/non-canonical) and positive or negative connotations. Their bias toward an association between canonical and positive is small and thus mostly affected by the re-learning of an assumed incongruent association such as non-canonical and positive and the process of “negative transfer.”

Nevertheless, we are aware of some limitations of our study. First, the group of hearers was not as homogenous as would have been ideal in the sense that there were differences between participants over which we had no control. Further research might highlight additional factors interacting with differences in IAT effects between individual listeners or listener groups. For example, it would have been interesting to also assess the listeners’ *explicit* attitudes toward French learners of German and of adolescent speakers from Kreuzberg with whom the tested variant is highly associated. Also, incorporating personality constructs such as openness – one of the dimensions of the Five Factor Model describing differences in personality (McCrae and John, 1992) – or the proximity of a listener to ideologies such as *conservatism* and *liberalism* (Kerlinger, 1984) – which reflect a person’s attitudes toward changes (personal or political) – might give insights into the reasons for differences in the sensitivity to priming effects and IAT effects and toward linguistic change in general.

Second, while we did look for an effect of lexical frequency of the test items on the absolute RTs, our material was not designed to investigate the effect of lexical frequency in a controlled manner. With our limited selection of stimuli, we did not find systematic variation. Nevertheless, it would be interesting to explore more the effect of lexical frequency of the different test items on the IAT-effect in the sense that more frequent words facilitate, and less frequent words inhibit the implicit association between concept categories and valence categories.

While we believe that there is a general lack of awareness that one phonetic variant belongs to a specific social group, our results strongly suggest that implicit associations are drawn between fine phonetic detail and social groups. Moreover, these associations are affected by listeners’ background, i.e., their attitudes, beliefs, stereotypes, and shared world knowledge pointing to language processing which needs to incorporate culturally and socially situated contexts.

## Data Availability Statement

The raw data supporting the conclusions of this article will be made available by the authors, without undue reservation, to any qualified researcher.

## Ethics Statement

The studies involving human participants were reviewed and approved by Ethics Committee of the DGfS (#2019-07-190625). Written informed consent was obtained from all participants. Written informed consent from the participants’ legal guardian/next of kin was not required to participate in this study in accordance with the national legislation and the institutional requirements.

## Author Contributions

The idea and conceptualization of the investigation, data analysis, and writing of the manuscript was joint work by SJ and MW. The execution of the experiment was done by GS and SJ. The data curation was carried out by GS. Visualization was conducted by MW. Funding acquisition and project administration was done by SJ. All authors contributed to the article and approved the submitted version.

## Conflict of Interest

The authors declare that the research was conducted in the absence of any commercial or financial relationships that could be construed as a potential conflict of interest.

## Footnotes

1 https://implicit.harvard.edu/implicit/germany/
